# From aging to Alzheimer’s disease: concordant brain DNA methylation changes in late life

**DOI:** 10.1186/s13073-026-01698-8

**Published:** 2026-06-16

**Authors:** David Lukacsovich, Juan I. Young, Lissette Gomez, Michael A. Schmidt, Wei Zhang, Brian W. Kunkle, Xi Steven Chen, Eden R. Martin, Lily Wang

**Affiliations:** 1 Division of Biostatistics, Department of Public Health Sciences, University of Miami, Miller School of Medicine, Miami, FL 33136 USA; 2 Dr. John T Macdonald Foundation Department of Human Genetics, University of Miami, Miller School of Medicine, Miami, FL 33136 USA; 3 John P. Hussman Institute for Human Genomics, University of Miami Miller School of Medicine, Miami, FL 33136 USA; 4 Sylvester Comprehensive Cancer Center, University of Miami, Miller School of Medicine, Miami, FL 33136 USA; 5 Soffer Clinical Research Ctr, University of Miami School of Medicine, Miami, USA 1120 NW 14th St, FL 33136

**Keywords:** Aging, Alzheimer’s disease, DNA methylation, Epigenetics, Biomarkers

## Abstract

**Background:**

Aging is the strongest risk factor for Alzheimer’s disease (AD), but the molecular connections between aging and AD remain unclear. DNA methylation (DNAm) is implicated in both processes.

**Methods:**

We conducted a meta-analysis of DNAm in prefrontal cortex from two independent postmortem cohorts: the Religious Orders Study and Memory and Aging Project (ROSMAP) and Brains for Dementia Research (BDR). Age-associated CpG sites were identified using cohort-specific linear models adjusted for neuronal proportion, sex, and batch, followed by meta-analysis. We computed epigenetic age acceleration in brain samples as delta-age (DNAmAge − chronological age), and compared clinically diagnosed AD with cognitively unimpaired participants. Functional analyses included genomic feature enrichment, pathway analysis, brain-blood DNAm correlation, and colocalization with genome-wide association study (GWAS) loci. Prognostic relevance of age-associated CpGs was tested in the Alzheimer’s Disease Neuroimaging Initiative (ADNI) dataset using Cox proportional hazards models for disease progression.

**Results:**

We identified 3264 CpG sites associated with aging; most were hypermethylated and enriched in promoters and CpG islands, and involved genes related to immune regulation and metabolism. Comparison with AD neuropathology-associated methylation showed substantial overlap, with nearly all shared CpGs and regions showing concordant directional changes. Cortical epigenetic age acceleration was higher in ROSMAP participants with clinical AD than in cognitively unimpaired individuals after covariates adjustment, and this association persisted when the cortical clock was restricted to the aging-associated CpGs identified here, suggesting that acceleration in AD is attributable to age-related CpGs. Several CpGs showed significant brain-blood methylation correlations or were linked to AD GWAS risk loci through colocalization analyses. In ADNI, among 33 candidate CpGs selected for concordant aging- and AD-associated changes in cortex and significant brain-blood methylation correlations, baseline methylation at one CpG (cg10752406 in *AZU1* promoter) was associated with progression at a 5% false discovery rate after covariate adjustment.

**Conclusions:**

Aging-associated DNAm changes in prefrontal cortex overlap with AD neuropathology-related changes and are involved in accelerated epigenetic aging in clinical AD. Our study provides valuable insights into the epigenetic landscape of aging and its implications for AD.

**Supplementary Information:**

The online version contains supplementary material available at 10.1186/s13073-026-01698-8.

## Background

Alzheimer’s disease (AD), the most common neurodegenerative disorder, is anticipated to affect nearly 13 million people by 2050 [1]. This rise in AD cases is expected to increase healthcare costs significantly, from $355 billion in 2021 to $1.1 trillion in 2050. Aging is the strongest risk factor for AD. The percentage of people with AD increases from 5% for 65–74-year-olds to 14% in 75–84-year-olds and 35% in people older than 85 [2, 3]. However, our current understanding of how age-related molecular processes contribute to AD is still very limited.

Epigenetic modifications, particularly DNA methylation (DNAm), play a crucial role in understanding aging and AD. It has been observed that as people age, DNAm decreases at intergenic regions but increases at many promoter-associated CpG islands regions [4]. Common age-associated changes across individuals have been observed at many loci [5–13]. DNAm also plays an important role in AD [14–18]. In Zhang et al. (2020), we identified methylation differences that were consistently associated with AD neuropathology in multiple brain EWAS [19]. Our analyses showed the majority of these AD neuropathology-associated DNAm differences in the brain were hyper-methylated with advancing AD Braak stage, many are located in CpG islands, and were significantly enriched in polycomb repressive regions and genes involved in the immune processes [19].

Although DNAm in aging has been previously described, their relevance to AD is less explored. Clearly, aging and AD are intertwined [20], and the exact contribution of age-related DNAm changes to AD remains to be clarified. Most previous studies of DNAm differences in AD treated age as a confounding variable to be adjusted for and did not discuss how AD-associated DNAm differences relate to those occurring in normal aging. In addition, while a recent study identified distinct risk factors for dementia in early (< 45 years), midlife (45–60 years), and late-life (≥ 65 years) [21], most DNAm studies in aging did not stratify by age group of the subjects in a particular period of life. Finally, most of the aging studies have utilized blood samples. However, for neurological disorders such as AD, the use of disease-relevant tissue (e.g., brain tissues) is often preferred for epigenetic studies.

In this study, we aimed to compare the changes in brain DNAm during normal aging with those occurring in AD in late life, a time when AD is most prevalent. To this end, we performed a meta-analysis of two large cohorts of postmortem prefrontal cortex brain samples from subjects over 65 years old, generated by the Religious Orders Study and Rush Memory and Aging Project (together as ROSMAP [22]) and the Brains for Dementia Research (BDR) study [23]. By comparing the age-associated DNAm changes with those previously identified to be associated with AD neuropathology, we identified a subset of DNAm alterations that are common in normal aging and AD neuropathology in the frontal cortex. Next, to evaluate the feasibility of these DNAm changes as biomarkers, we performed colocalization analysis incorporating results from recent dementia genome-wide association studies (GWAS), assessed brain-to-blood DNAm correlations at these CpGs, and performed an out-of-sample validation using an external longitudinal AD progression dataset. Our findings offer valuable insights into the DNAm changes in aging and their implications for AD.

## Methods

### Study cohorts

Our study used publicly available DNAm datasets from postmortem brain tissue and matched brain-blood samples, together with an independent longitudinal blood DNAm dataset for validation (Additional file 1: Table S1). The primary discovery analysis was based on prefrontal cortex DNAm profiles from two independent postmortem brain cohorts, ROSMAP and BDR. ROSMAP comprises two longitudinal clinicopathologic cohort studies of aging and dementia in which older adults without known dementia at enrollment agree to annual clinical evaluations and brain donation at death [24]. BDR is a United Kingdom brain-bank network that recruits older adults with and without neurodegenerative disease for prospective clinical and cognitive assessments during life, with consent for standardized postmortem brain donation and neuropathologic characterization [25]. DNAm data for ROSMAP and BDR were obtained from the AD Knowledge Portal [26] and the Gene Expression Omnibus database [27], respectively.

For the primary meta-analysis of age-associated DNAm in late life, we included samples from participants older than 65 years with minimal or absent AD neuropathology. In ROSMAP, we selected prefrontal cortex samples with neurofibrillary tangle Braak stages 0-II. We excluded samples from Black participants (*n* = 2), participants of Hispanic origin (*n* = 2), and samples with postmortem interval (PMI) greater than 72 h (*n* = 4) [28]. In BDR, neuropathologic Braak scores were not available to us. Therefore, we used a DNAm-based Braak prediction approach based on previously described cell-type methylation reference panels and retained samples classified as low-pathology, corresponding to predicted Braak stages 0-II. After sample selection and quality control, the primary aging meta-analysis included 332 prefrontal cortex DNAm profiles from ROSMAP and BDR. The average ages at death were 83.02 ± 6.09 years in ROSMAP and 80.25 ± 7.44 years in BDR, and approximately 51% of participants in each cohort were male (Additional file 1: Table S2).

The ROSMAP and BDR prefrontal cortex samples were used for the primary CpG-level and region-based meta-analyses of DNAm associated with age at death. The same primary meta-analysis results were used for genomic feature enrichment, chromatin-state enrichment, pathway analysis, comparison with previously reported AD Braak stage-associated DNAm differences, and integration with brain methylation quantitative trait loci and Alzheimer’s disease and related dementias (ADRD) GWAS summary statistics.

Additional datasets were used for secondary and validation analyses. For cortical epigenetic age acceleration, we used ROSMAP prefrontal cortex samples with available clinical diagnosis at death and cortical clock CpGs after quality control. This analysis included 536 ROSMAP participants, including 307 with clinical AD and 229 cognitively unimpaired controls. The mean age at death was 87.78 ± 3.55 years for AD cases and 84.42 ± 5.49 years for cognitively unimpaired controls. To evaluate whether age acceleration was also present in a brain region relatively spared by AD pathology, we analyzed cerebellum DNAm data from three independent GEO datasets (GSE134379 [29], GSE105109 [30], and GSE59685 [31]), which included cerebellar DNAm profiles from AD cases and control or non-demented individuals from independent postmortem brain resources, including the Banner Sun Health Research Institute Brain and Tissue Bank and the MRC London Brain Bank. We computed cerebellar delta-age within each dataset and fitted dataset-specific linear models, followed by inverse-variance-weighted fixed-effects meta-analysis.

For brain-to-blood DNAm correlation analyses, we used the London cohort, which included 69 individuals with matched prefrontal cortex and blood DNAm data [32]. These analyses evaluated whether aging-associated CpGs identified in brain also showed correlated DNAm levels in blood after adjustment for age, sex, batch effects, and estimated cell-type proportions.

For out-of-sample validation of candidate peripheral biomarkers, we used data from the Alzheimer’s Disease Neuroimaging Initiative (ADNI) study, an independent longitudinal cohort with whole-blood DNAm data and follow-up clinical diagnosis [33]. We analyzed the earliest available blood DNAm sample for each participant and evaluated whether candidate CpGs were associated with disease progression, defined as conversion from cognitively unimpaired (CU) to mild cognitive impairment (MCI) or AD, or from MCI to AD. The ADNI validation analysis included 538 participants with available DNAm and follow-up data.

### Pre-processing of DNA methylation data

The samples in the ROSMAP and BDR study were measured using the Infinium HumanMethylation450 [34] and Infinium MethylationEPIC BeadChip [35], respectively. The numbers of excluded probes and samples at each quality control (QC) step are summarized in Additional file 1: Table S3. Probe QC removed CpGs with detection *P*-value > 0.01 in any sample within a cohort using minfi [36], cross-reactive probes [37], probes with a SNP of minor allele frequency (MAF) > 0.01 within the final 5 bases of the probe, and probes on sex chromosomes. This filtering was implemented with DMRcate using rmSNPandCH with dist = 5 and mafcut = 0.01 [38].

Sample QC excluded arrays with bisulfite conversion efficiency below 85% and samples for which recorded sex disagreed with DNAm-predicted sex. After QC, methylation beta values were normalized using a QN.BMIQ workflow [39]. Briefly, quantile normalization from the lumi R package [40] was applied to reduce sample-level systematic variation, followed by BMIQ normalization in wateRmelon [41, 42] to adjust Infinium type I and type II probe distributions. For each cohort, principal component analysis (PCA) of the 50,000 most variable CpGs was used to identify outliers; samples more than 3 standard deviations (SDs) from the mean of either of the first two principal components were removed.

Detailed sample inclusion criteria for ROSMAP and BDR are described above under Study cohorts. Specifically, for BDR, the low-pathology classification used DNAm reference panels for NeuN + (neuronal-enriched), SOX10 + (oligodendrocyte-enriched), and NeuN–/SOX10– (microglia/astrocyte-enriched) cell populations developed by Shireby et al. [43], together with the Houseman deconvolution algorithm [44], to estimate cell-type proportions in bulk cortex. We applied k-means clustering (k = 3) to these estimated cell-type proportions and retained the cluster corresponding to predicted Braak stages 0-II (Additional file 2: Fig. S1), yielding 196 BDR samples, as described in Smith et al. [45]. In the BDR dataset, the ages of the brain samples were estimated using the cortical clock method, which was shown to accurately predict age in the human brain cortex for BDR samples [23].

### Statistical analysis of individual CpGs

CpG-level analyses were first performed separately within each cohort. For each CpG, we modeled methylation M-values as the outcome and age at death as the primary predictor, because M-values provide more appropriate distributional properties for linear modeling than beta values [46]. ROSMAP and BDR models adjusted for sex, batch, methylation plate, and neuronal proportion estimated with CETS [47]. PMI was unavailable in BDR. In ROSMAP, PMI was not correlated with age at death (r = 0.0033, *P*-value = 0.9691), so it was not included as a covariate.

### Inflation assessment and correction

We evaluated and corrected test-statistic inflation with bacon [48], a method designed for EWAS and other high-dimensional association studies in which many loci may be truly associated. Bacon estimates an empirical null from a three-component normal mixture model of the CpG-wise test statistics; the null mean represents bias and the null SD represents inflation. For single-CpG analyses, the uncorrected lambda values were 1.12 in ROSMAP and 1.18 in BDR, with estimated biases of −0.13 and −0.17. After bacon correction, the corresponding bias estimates were 1.05 × 10^–3^ and −3.40 × 10^–4^, and lambda values were 1.00 in both cohorts. For region-level analyses, lambda values were 1.14 and 1.23 before correction and 1.00 and 1.01 after correction; bias estimates changed from −0.19 and −0.09 before correction to −2.33 × 10^–3^ and 1.34 × 10^–2^ after correction.

### Meta-analysis

Bacon-corrected cohort-specific effect estimates, standard errors, and *P*-values were combined using inverse-variance weighted fixed-effect meta-analysis implemented in the meta R package [49]. We controlled multiple testing with the Benjamini–Hochberg the false discovery rate (FDR) [50]. CpGs were considered significant if they had FDR < 0.05 in the meta-analysis and showed the same direction of effect in both cohorts.

### Region-based analysis

DMRs associated with age at death were identified using two complementary methods, comb-p [51] and coMethDMR [52], and only regions supported by both methods were retained. Comb-p was run on meta-analysis *P*-values to identify spatial clusters of low *P*-values, using –seed 1e-3 and –dist 200. Regions with Sidak-adjusted *P*-value < 0.05 were considered significant if they contained at least three CpGs and all CpGs in the region showed the same direction of age association.

For coMethDMR, we first defined contiguous genomic regions as sets of array CpGs separated by no more than 200 base pairs between neighboring probes. Within these regions, co-methylated subregions were selected and summarized by the median methylation M-value. Each subregion was then tested for association with age at death using linear regression adjusted for sex, estimated cell composition, methylation plate, and batch. Cohort-specific coMethDMR test statistics were bacon-corrected [48] and meta-analyzed with inverse-variance weighting using the meta R package. Co-methylated regions with at least three CpGs and meta-analysis FDR < 0.05 were considered significant. Downstream analyses focused on DMRs identified by both comb-p and coMethDMR.

### Functional annotations and enrichment analyses

Significant CpGs and DMR CpGs were annotated with Illumina annotations and GREAT [53] using default settings. We tested enrichment or depletion across genomic annotations (island, open sea, shelf, shore, 1stExon, 3'UTR, 5'UTR, body, intergenic, TSS1500, and TSS200) and Roadmap chromatin states using two-sided Fisher's exact tests. The 15-state chromatin annotations were obtained from Roadmap Epigenomics [54] for dorsolateral prefrontal cortex (E073), generated with ChromHMM [55]. Because 26 genomic or chromatin features were evaluated, *P*-value < 1.92 × 10^–3^ (0.05/26) was used as the significance threshold.

For feature-enrichment tests of individual CpGs, the foreground was the set of significant CpGs assigned to a given feature and the background was all tested CpGs assigned to that feature. For DMR analyses, the foreground was the set of CpGs within significant DMRs that mapped to a given feature, and the background was the set of CpGs in the array-based contiguous regions used for DMR discovery. This design ensured that enrichment was evaluated relative to the CpGs eligible for each analysis.

We used LOLA (Locus Overlap Analysis) software [56] to test whether aging-associated CpGs or DMRs overlapped transcription-factor and chromatin-protein binding sites more often than expected. Pathway enrichment was performed with missMethyl [57] using the union of significant single CpGs and CpGs located in significant DMRs, thereby avoiding double counting of genes represented by both CpG- and region-level signals. For all enrichment analyses, both foreground and background sets were restricted to CpGs present on both the 450 k array used for ROSMAP and the EPIC array used for BDR after QC.

### Comparison with AD Braak stage-associated DNA methylation differences

The significant AD Braak stage-associated CpGs and DMRs were obtained from Supplementary Data 15 and 2, respectively, in Zhang et al. [19]. The significant results from the matched samples analysis were obtained from Supplementary Data 6 and 7 in Zhang et al. [19]. The enrichment analysis results of AD Braak stage-associated CpGs and DMRs were from Supplementary Data 3 and 4 in Zhang et al. (2020). Both the analyses by Zhang et al. (2020) and this manuscript used the same DNAm data processing and inflation correction procedures, making the results highly comparable.

### Epigenetic age acceleration in AD

For cortical samples, delta-age was defined as delta-age = DNAmAge—chronological age, where DNAmAge was computed using cortical clock coefficients [23]. To determine if age acceleration was significantly higher in AD subjects, we applied a linear model with delta-age as the outcome, AD status as the main independent variable, and included covariates age at death, sex, estimated neuronal proportion, batch, and methylation plate. AD status was defined by consensus clinical diagnosis at time of death in ROSMAP study.

To evaluate whether observed differences in age acceleration were attributable to the aging-associated CpGs identified in this study, we recomputed DNAmAge using only cortical-clock CpGs that overlapped the 3264 FDR-significant aging-associated sites from our meta-analysis. This restriction yielded 100 CpGs. DNAmAge, delta-age, and the regression analysis were then repeated as described above.

To evaluate whether brain regions that are relatively spared by AD pathology also show age acceleration, we performed similar analyses in cerebellar tissue. DNAmAge in cerebellum was computed using a cerebellum-specific epigenetic clock [58]. For each of the three independent datasets with cerebellar methylation data, we defined cerebellar delta-age as DNAmAge.cerebellum—chronological age and fitted linear regression models with cerebellar delta-age as the outcome, AD status as the main predictor, and covariates age at death, sex, estimated neuronal proportion, and methylation plate. Effect estimates and standard errors for the AD term were then combined across datasets using an inverse-variance-weighted fixed-effects meta-analysis.

### Correlation and colocalization with genetic susceptibility loci

Brain methylation quantitative trait loci (mQTLs) for aging-associated CpGs, including significant single CpGs and CpGs in significant DMRs, were queried from the xQTL database [59], which provides Bonferroni-corrected brain mQTL associations (http://mqtldb.godmc.org.uk/downloads). SNP-CpG pairs were treated as cis associations when the SNP was within 5 kb of the CpG. ADRD GWAS summary statistics from Bellenguez et al. [60] were downloaded from the EBI GWAS Catalog [61] under accession GCST90027158. Colocalization between mQTL and ADRD association signals was tested with coloc [62] using default settings.

### Correlations between DNA methylation in blood and brain samples

In the London matched brain-blood dataset [32], we assessed adjusted Spearman correlations between blood and brain methylation for significant aging-associated CpGs and CpGs in aging DMRs. To remove major sources of confounding, M-values were residualized separately in brain and blood using linear models that included age, sex, methylation plate, and estimated cell-type proportions (neuronal proportion for brain and blood cell fractions for blood). Spearman correlations were then calculated between the resulting brain and blood residuals.

### Validation of concordant brain DNAm differences in aging and AD neuropathology

We used MIAMI-AD (https://miami-ad.org/) [63] to examine whether CpGs showing concordant associations with both aging and AD had been reported in prior AD-related DNAm studies. The CpG Query tool was used to search the phenotype categories “AD Biomarker”, “AD Neuropathology”, “Dementia Clinical Diagnosis”, and “Mild Cognitive Impairment (MCI)”. To maintain independence from the discovery analysis, results from ROSMAP and BDR were excluded from this comparison.

We then tested whether the concordant CpGs in aging and AD predicted clinical progression in ADNI. Cox proportional hazards models were fit with the survival R package, with progression status and follow-up time as the survival outcome and CpG beta value as the predictor of interest. Models adjusted for age, sex, *APOE* ε4 status, years of education, and baseline MMSE score: Surv(conversion event, follow-up time) ~ cpg.beta.value + age + sex + *APOE* ε4 status + years of education + baseline MMSE score. FDR was calculated across the 33 candidate CpGs. Covariate-adjusted survival curves from the multivariable Cox model were generated with survminer R package.

## Results

### DNA methylation differences are significantly associated with aging at individual CpGs and co-methylated regions

**Fig. 1:**
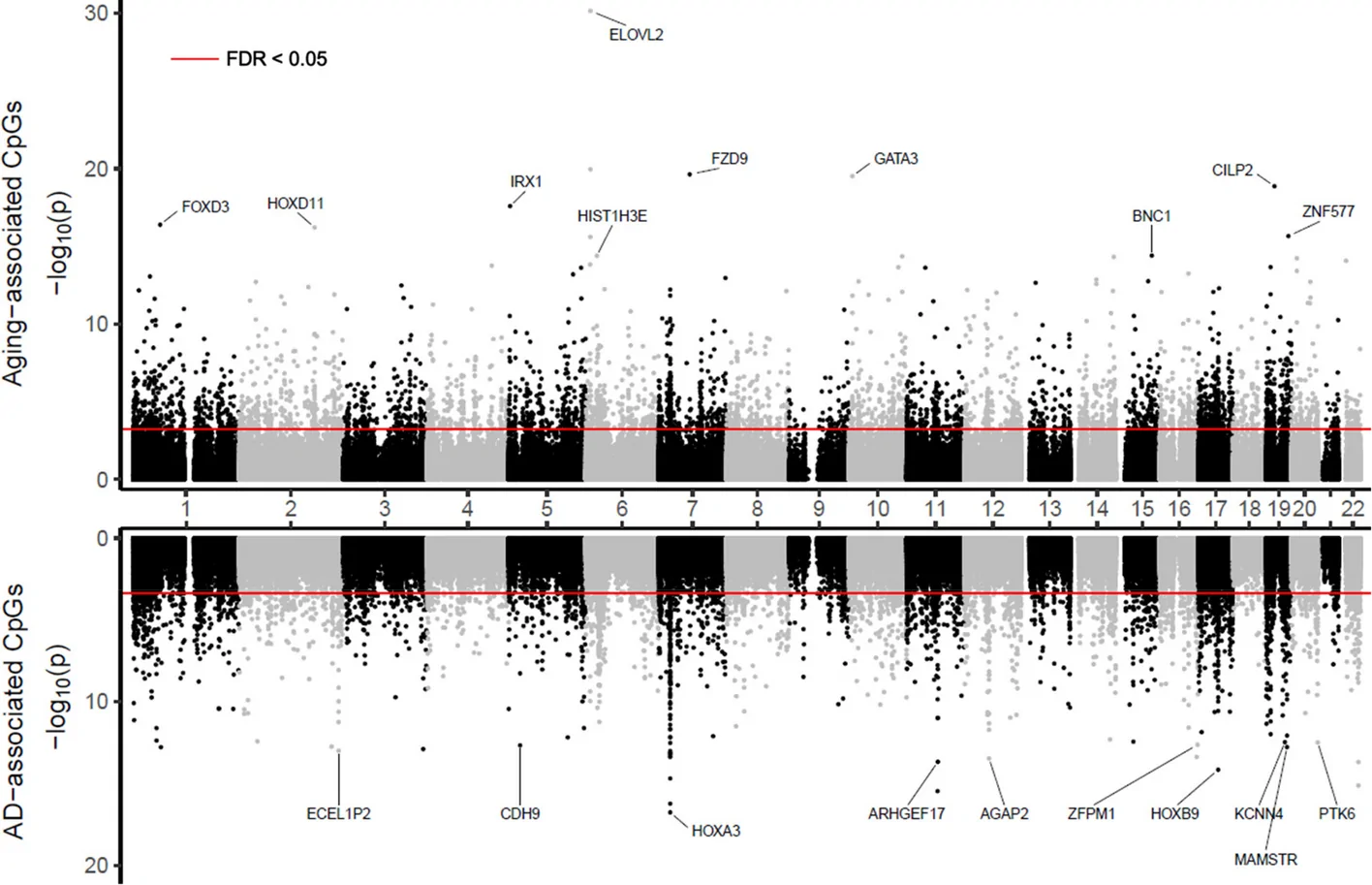
Miami plot of DNA methylation differences associated with aging and Alzheimer’s disease (AD) neuropathology in brain samples. The x-axis are chromosome numbers. The y-axis shows -log_10_(*P-*value) of the CpG to age-at-death associations (above the x- axis) or CpG to Braak stage associations (below the x-axis). Red lines indicate significance threshold at 5% false discovery rate

After quality control, the discovery meta-analysis included 332 prefrontal cortex DNA methylation profiles from ROSMAP and BDR participants older than 65 years with minimal or absent AD neuropathology, as described in Methods and summarized in Additional file 1: Table S2. Adjusting for estimated cell-type proportions (i.e., the proportion of neurons), sex, and batch effects, our meta-analysis of individual CpGs identified 3264 significant age-associated CpGs at a 5% false discovery rate (FDR) and with a consistent direction of change in both ROSMAP and BDR cohorts (Fig. 1, Additional file 1: Table S4). Among them, 394 CpGs also reached the 9 × 10^–8^ genome-wide significance level [64]. Consistent with previous studies [65, 66], we observed the majority of the significant CpGs (84.83%, 2769 CpGs) were hypermethylated with an increase in age.

Among the top 10 most significant CpGs (Table 1), three CpGs, including the two most significant ones, are located in the promoter region of the *ELOVL2* gene, where DNA methylation in this region was shown to be one of the most robust markers of aging in various tissues, including both brain and blood [67–69]. Interestingly, among the 3264 FDR-significant CpGs, 134 CpGs (4.10%), all of which are hypermethylated in aging, are located on genes belonging to the *HOX* family. This is significantly higher than the overall proportion of probes targeting *HOX* genes (1251 *HOX* gene probes among 740,555 tested probes, or 0.15%, *P-*value = 2.2 × 10^–16^). DNAm and gene expression changes in *HOX* genes have been reported during aging in various cell types, such as human hemopoietic cells, dermal cells, and muscle-derived cells [70–72]. The *HOX* genes encode transcription factors that determine cellular identity and ensure that stem cells can properly differentiate into various cell types needed to maintain tissue function and repair [73, 74]. These processes involve the continuous renewal, repair, and replacement of cells within a tissue to preserve proper tissue function.

**Table 1:**
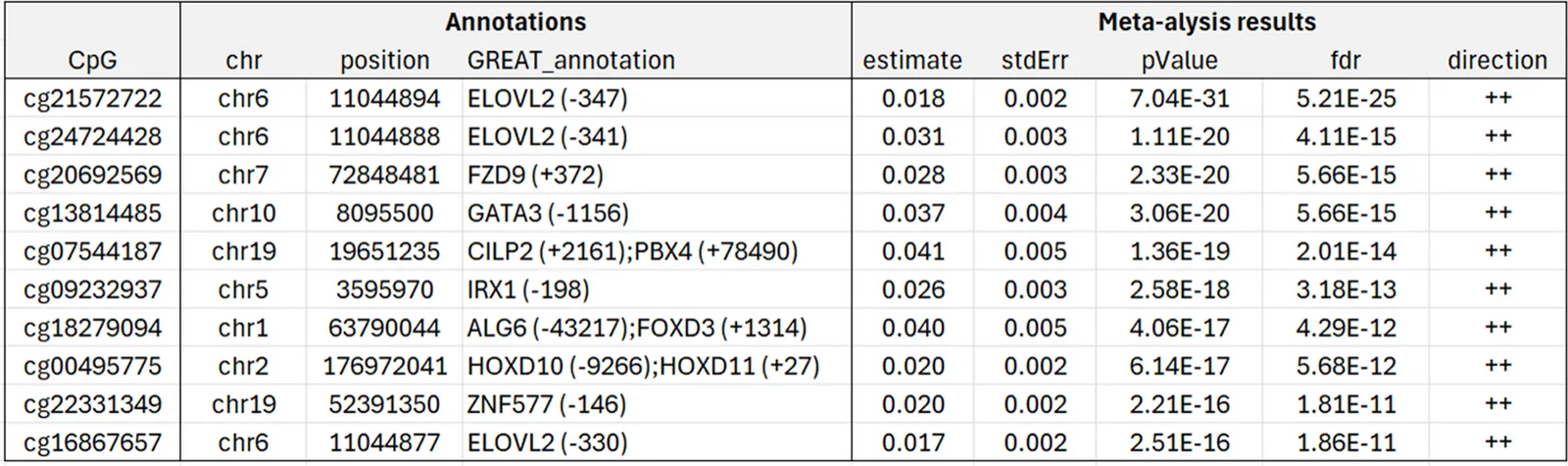
Top 10 most significant CpGs associated with age at death in meta-analysis of ROSMAP and BDR datasets. For each CpG, annotations include the location of the CpG (Chr, Position), nearby genes based on GREAT software. The inverse-variance weighted meta-analysis regression model results include estimated effect size (estimate) where CpGs that are hyper-methylated with increased age have positive values, standard error (stdErr), *P*-value (pValue), and false discovery rate (fdr) for multiple comparison corrections. The last column (direction) indicates the direction of effects in ROSMAP and BDR cohorts. All P-values are two-sided. In GREAT annotation, the numbers in parentheses indicate distance from the TSS

In region-based analysis, after multiple comparisons correction, comb-p software [51] identified 191 significant DMRs associated with age at death at 5% Sidak adjusted *P-*value. Among them, 69 DMRs were also identified by the coMethDMR software [52] at 5% FDR (Additional file 1: Table S5). For these 69 DMRs, the average number of CpGs per DMR is 4.86 ± 6.23 CpGs. As with significant CpGs, the majority of the DMRs (86.96%, 60 DMRs) were also hypermethylated with increased age. Moreover, the most significant DMR is located in the *ELOVL2* gene, and five of the top 10 most significant DMRs are located in the *HOX* genes. Among other genes associated with the top 10 DMRs (Table 2), the *ISM1* gene encodes a secreted protein that is widely distributed across various body compartments and is involved in numerous biological processes in aging, including angiogenesis (formation of new blood vessels), metabolism, and immune responses [75, 76]. Our observed hypermethylation in the promoter region of the *ISM1* gene is consistent with a previous study that demonstrated *ISM1* gene expression decreases with age and nominated this gene as a potential biomarker for aging [77]. The *KLF14* gene encodes a zinc finger-containing transcription factor. A recent study demonstrated that *KLF14* expression decreases with aging in various tissues, including blood, liver, and peripheral blood lymphocytes, and this decrease is associated with accelerated cellular senescence and aging-related pathologies [78]​​. These findings are consistent with our observed hypermethylation in the promoter region of the *KLF14* gene associated with increased age.

**Table 2:**
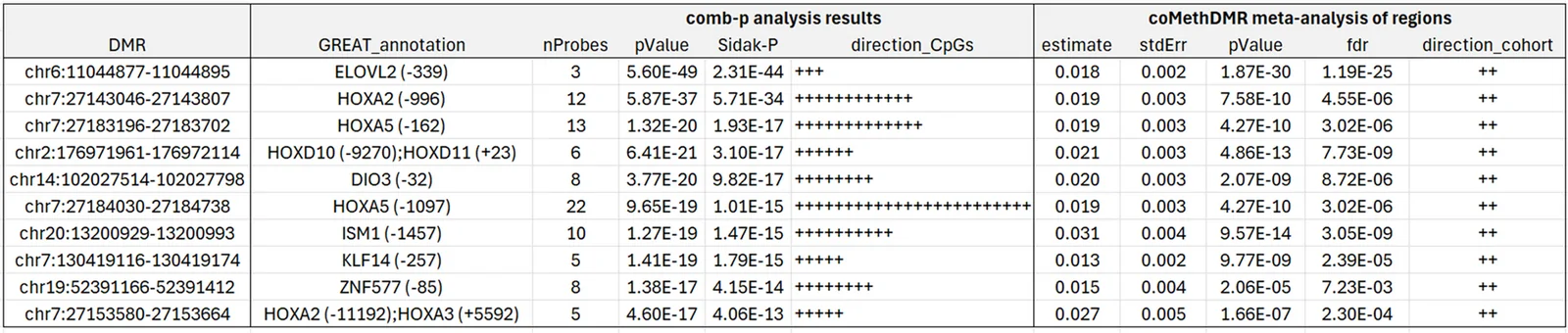
Top 10 most significant differentially methylated regions (DMRs) associated with age at death identified by both comb-p and coMethDMR software in meta-analysis. For each DMR, annotations include location of the DMR (DMR) and nearby genes based on GREAT. Comb-p results include the number of probes (nProbes), multiple comparison corrected P-value based on Sidak method (Sidak P), and direction of each CpG within the DMR (direction_CpGs). The results based on coMethDMR include estimated effect size (estimate) and standard error (stdErr), pValue, false discovery rate (fdr) from inverse-variance weighted meta-analysis regression model, and direction of effect size in each cohort (direction_cohort). All *P*-values are two-sided. In GREAT annotation, the numbers in parentheses indicate distance from the TSS

### Enrichment analysis highlighted the potential functional relevance of the aging-associated DNA methylation differences

**Fig. 2:**
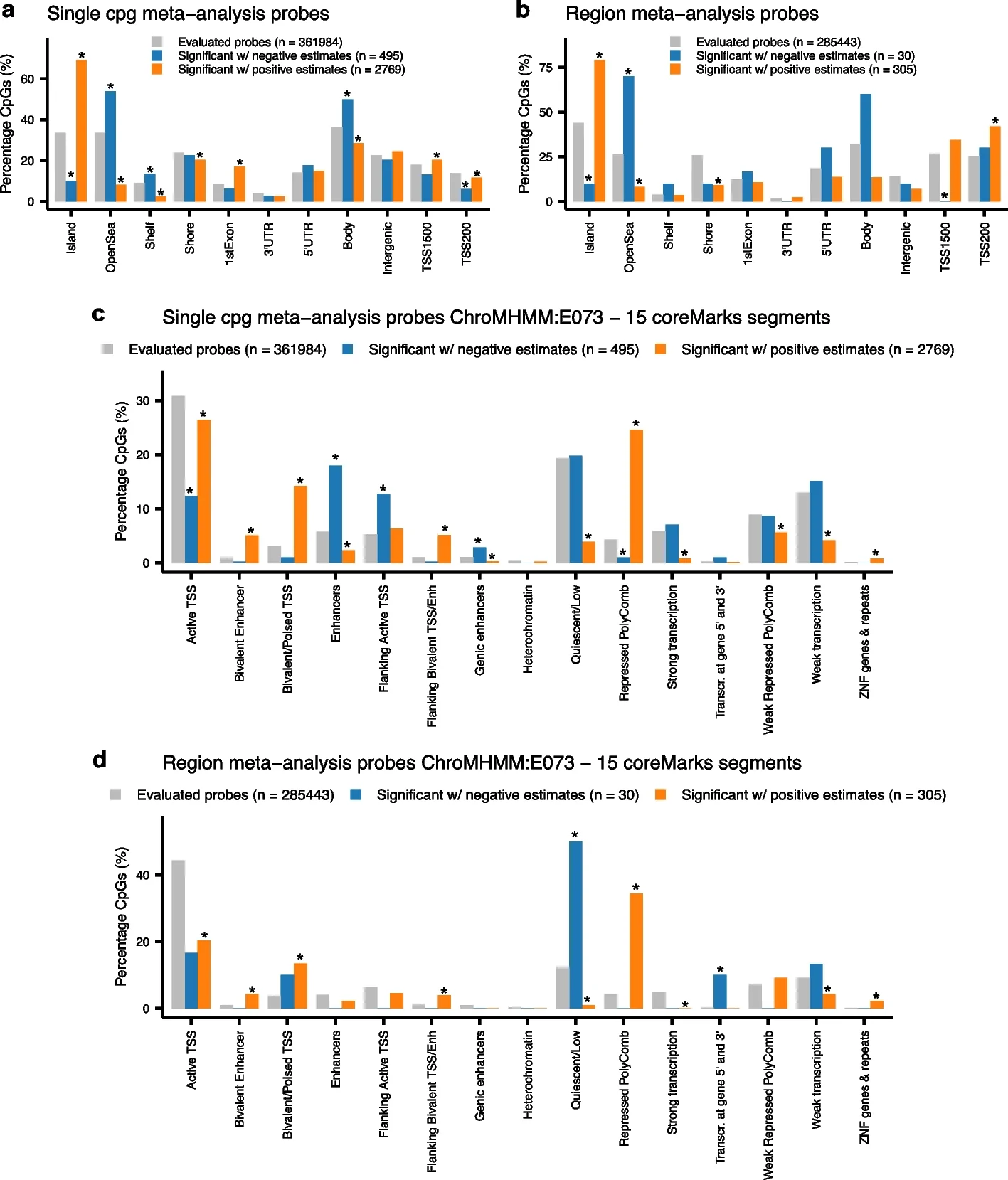
Enrichment of DNA methylation significantly associated with chronological age in meta-analysis of individual CpGs and DMRs. A two-sided Fisher’s test was used to determine over or under-representation of the significant CpGs in individual CpGs analysis (**a** and **c**) and CpGs mapped within significant DMRs (**b** and **d**) in various genomic features (**a** and **b**) and chromatin states (**c** and **d**). Because a total of 26 genomic features (11 genomic locations and 15 chromatin states), we considered *P-*values less than 1.92 × 10^–3^ (i.e., 0.05/26) to be statistically significant **a** and **c** - individual CpGsb and **d** - DMRsa and **b** - genomic featuresc and **d** - chromatin states

We next tested the enrichment of the aging-associated methylation differences against various genomic features. Both significant hypermethylated individual CpGs and DMRs were enriched in *CpG island*, but depleted in the *open sea*, *shore*, and *gene body* (Additional file 1: Tables S6-S7, Fig. 2a-b). There were also slight differences in the enrichment of hypermethylated CpGs and DMRs. The significant hyper-methylated individual CpGs were over-represented in * 1 st exon*, and *TSS1500*, but under-represented in *TSS200* and *shelf*. On the other hand, the hyper-methylated DMRs were enriched in *TSS200*, but under-represented in *intergenic* regions. In contrast, both significant hypo-methylated individual CpGs and DMRs were enriched in the *open sea* but depleted in *CpG islands*. The hypo-methylated CpGs were additionally enriched in *shelf*, and *gene body*, and under-represented in *TSS200*, while the hypo-methylated DMRs were under-represented in *TSS1500*.

In addition, we also compared our results with chromatin states generated by the Roadmap Epigenomics project [54, 79]. Our enrichment analysis showed that significant hyper-methylated DMRs and CpGs were both enriched in regions with potential regulatory activity but are kept in a poised state (*bivalent enhancer*, *bivalent/poised TSS*, *flanking bivalent TSS/Enh*, *repressed polycomb*, and *ZNF genes & repeats*), but under-represented in regions with either active transcription (*active TSS*, *strong transcription*) or low level of transcription (*quiescent/low*, *weak transcription*) regions (Additional file 1: Tables S8-S9, Fig. 2c-d).

For hypomethylation in aging, there was less agreement for the enrichment of significant individual CpGs and DMRs. Specifically, the hypomethylated CpGs were enriched in *flanking active transcription start sites (TSSs), genic enhancers,* and *enhancers* which are distal regulatory elements that increase the transcription of target genes, but were depleted in *active TSS* and *repressed polycomb* regions. On the other hand, the hypo-methylated DMRs were enriched in *quiescent/low* and *transcription at gene 5' and 3'* regions associated with transcription initiation and termination.

Similarly, enrichment tests for regulatory elements using the LOLA software [56] also supported the potential functional relevance of these significant DNAm differences. In particular, age-associated hypermethylation were enriched in the binding sites of numerous transcription factors and chromatin proteins assayed by the ENCODE project [80] (Additional file 1: Table S10). In contrast, age-associated hypomethylation showed little to no enrichment, with only a single isolated significant signal.

Among the top hits were EZH2 and SUZ12, which are subunits of the polycomb repressive complex 2 (PRC2) protein complex. This finding is consistent with the observed enrichment of DNAm differences in PRC2 repressed regions and the CpG islands where PRC2 often binds (Fig. 2c-d), as well as previous observations that DNAm often interacts with PRC2 binding [81–83]. We also found strong enrichment at binding sites of CTCF, a transcription factor essential for regulating gene expression and chromatin architecture by acting as an insulator. Intriguingly, aging-associated DNAm changes in blood have been consistently observed to be enriched at CTCF binding sites across multiple platforms for measuring DNA methylation [12, 84, 85]. These DNAm at CpG sites within CTCF motifs can antagonize the binding of the CTCF protein, thereby affecting chromatin architecture and gene expression. It has been estimated as much as 40% of the variability in CTCF binding is attributed to DNAm [86]. Other significant transcription factors and chromatin proteins also play important roles in aging by regulating various critical processes such as cellular metabolism (CtBP2, GR), development (CtBP2, Max, TCF12, TEAD4, NANOG), apoptosis (CtBP2, NRSF, Max), chromatin structure (CHD1, HDAC2, SETDB1), DNA repair (Rad21), and gene expression (HDAC2, SETDB1, TCF7L2, TEAD4, NANOG).

Finally, the Gene Ontology (GO) analysis of aging-associated DNA methylation differences highlighted the extensive influence of aging (Additional file 1: Table S11). Developmental processes, including embryonic and organ development, neural differentiation, and morphogenesis, are significantly enriched, indicating that aging impacts fundamental growth and differentiation pathways. The significant enrichment in cell fate specification and differentiation processes further suggests that aging is associated with the ability of cells to develop into specialized types, which is critical for tissue maintenance and repair. Additionally, the regulation of biological processes and communication, such as those involving blood circulation, points to age-related changes in physiological regulation and cell communication.

### Concordant DNA methylation differences associated with both aging and Alzheimer's disease neuropathology show a consistent direction of effect and common regulatory regions with enrichment

**Fig. 3:**
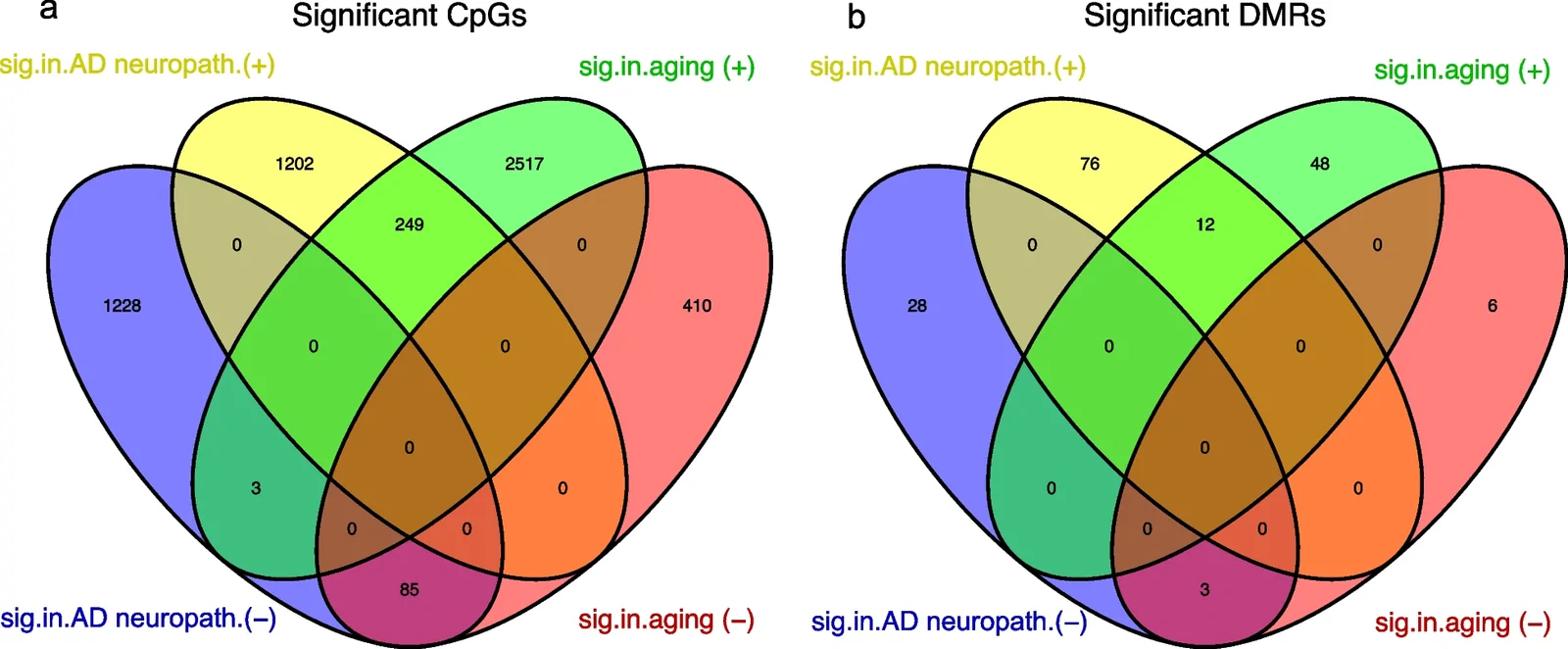
Almost all of the CpGs and DMRs significantly associated with both age and AD Braak stage showed concordant direction of change. In (**a**), among the overlapping CpGs, 249 out of 252 CpGs that showed hypermethylation with increasing age also showed hypermethylation with advancing Braak stage, and 85 out of 85 CpGs that showed hypomethylation with increasing age also showed hypomethylation with advancing Braak stage. Similarly, in (**b**), all 15 overlapping DMRs have concordant direction of change in aging and AD Braak stage. The significant AD Braak- associated DNAm were obtained from Supplementary Data 2 and 15 in Zhang et al. (2020) Nat. Comm. (PMID: 33257653). Abbreviations sig.in.aging (+): significantly hypermethylated in aging; sig.in.aging (-): significantly hypomethylated in aging; sig.in.AD neuropath. (+): significantly hypermethylated with increased AD Braak stage; sig.in.AD neuropath. (-): significantly hypomethylated with increased AD Braak stage

To further understand the methylation patterns in aging relative to Alzheimer’s disease neuropathology, we compared the significant aging-associated CpGs and DMRs in this study (Additional file 1: Tables S4-S5) with significant AD Braak stage-associated CpGs adjusting for age we previously identified in Zhang et al. [19] using the same DNA methylation processing pipeline and inflation correction procedures. We found that while the overwhelming majority (84.83%, 2769 out of 3264 CpGs) of aging-associated CpGs are hypermethylated, only slightly more than half (52.43%, 1451 out of 2767 CpGs) of the AD Braak stage-associated CpGs are hypermethylated. However, among the CpGs that are significant with both aging and AD neuropathology, almost all have concordant directions of change. Specifically, among the overlapping CpGs, almost all CpGs (249 out of 252 CpGs) that showed hypermethylation with increasing age also showed hypermethylation with advancing Braak stage. Moreover, all CpGs (85 out of 85 CpGs) that showed hypomethylation with increasing age also showed hypomethylation with advancing Braak stage (Fig. 3a, Additional file 1: Table S12). Furthermore, among the 69 aging-associated DMRs, 15 are significantly associated with AD Braak stage, and all 15 DMRs have concordant direction of change in aging and AD Braak stage (Fig. 3b, Additional file 1: Table S13). In Zhang et al. (2020), we also performed a matched samples analysis, in which each pathological AD case was paired with a control of the same sex and age at death within the same cohort, so that the DNAm differences from this analysis could be more confidently attributed to AD pathology rather than to confounding factors related to age or sex. The comparison of aging-associated CpGs and DMRs with AD Braak stage-associated DNAm in the matched analysis revealed a consistent pattern—all the DNAm differences significantly associated with both age (at death) and AD Braak stage showed concordant effect sizes in the same direction (Additional file 2: Fig. S2, Additional file 1: Tables S14-S15).

In addition, we also compared enrichment analysis results of aging-associated DNAm with those of AD Braak-associated DNAm. Interestingly, we found a few discordant genomic features: while aging-associated CpGs are depleted in CpG island shores, gene bodies, and enhancers, AD Braak-associated CpGs are enriched in genomic regions with these features (Additional file 1: Tables S6 and S8). However, the majority of the enrichment results are consistent and showed common regulatory regions. In both aging and AD neuropathology, the hypermethylated CpGs were enriched in CpG islands, but under-represented in open sea and shelf regions (Fig. 2a-b, Additional file 1: Table S6). Across the different chromatin states, in both aging and AD neuropathology, the hypermethylated CpGs were enriched in bivalent enhancers and repressed polycomb regions but depleted in active TSS and both quiescent/low and strong transcription regions (Fig. 2c-d, Additional file 1: Table S8). Taken together, these results highlighted the strong concordance in methylation changes (both hypermethylation and hypomethylation) between aging and AD neuropathology for DNAm that are significant in both analyses, underscoring common regulatory changes in both aging and AD neuropathology.

### Epigenetic age acceleration in AD

Using the ROSMAP prefrontal cortex samples described in Methods, we applied the cortical epigenetic clock [23] to test whether AD is associated with accelerated epigenetic aging beyond the overlap between age- and AD neuropathology-associated CpGs. Among the 347 probes in the cortical clock, 331 probes passed QC and were available in the ROSMAP DNAm dataset. For each subject, we computed delta-age (delta-age = DNAmAge—chronological age) and modeled delta-age as a function of clinical AD diagnosis (AD vs. cognitively unimpaired), adjusting for age at death, sex, neuronal proportion, batch, and methylation sample plate. We found that subjects with AD had significantly higher cortical delta-age than CU subjects (estimate = 1.47, *P-*value = 7.78 × 10^–5^), which corresponds to approximately 1.5 years of additional epigenetic age acceleration after covariate adjustment. This finding extends prior studies that reported significant associations between epigenetic age acceleration and AD neuropathology in the cortex in ROSMAP [87, 88] to clinical AD cases.

**Fig. 4:**
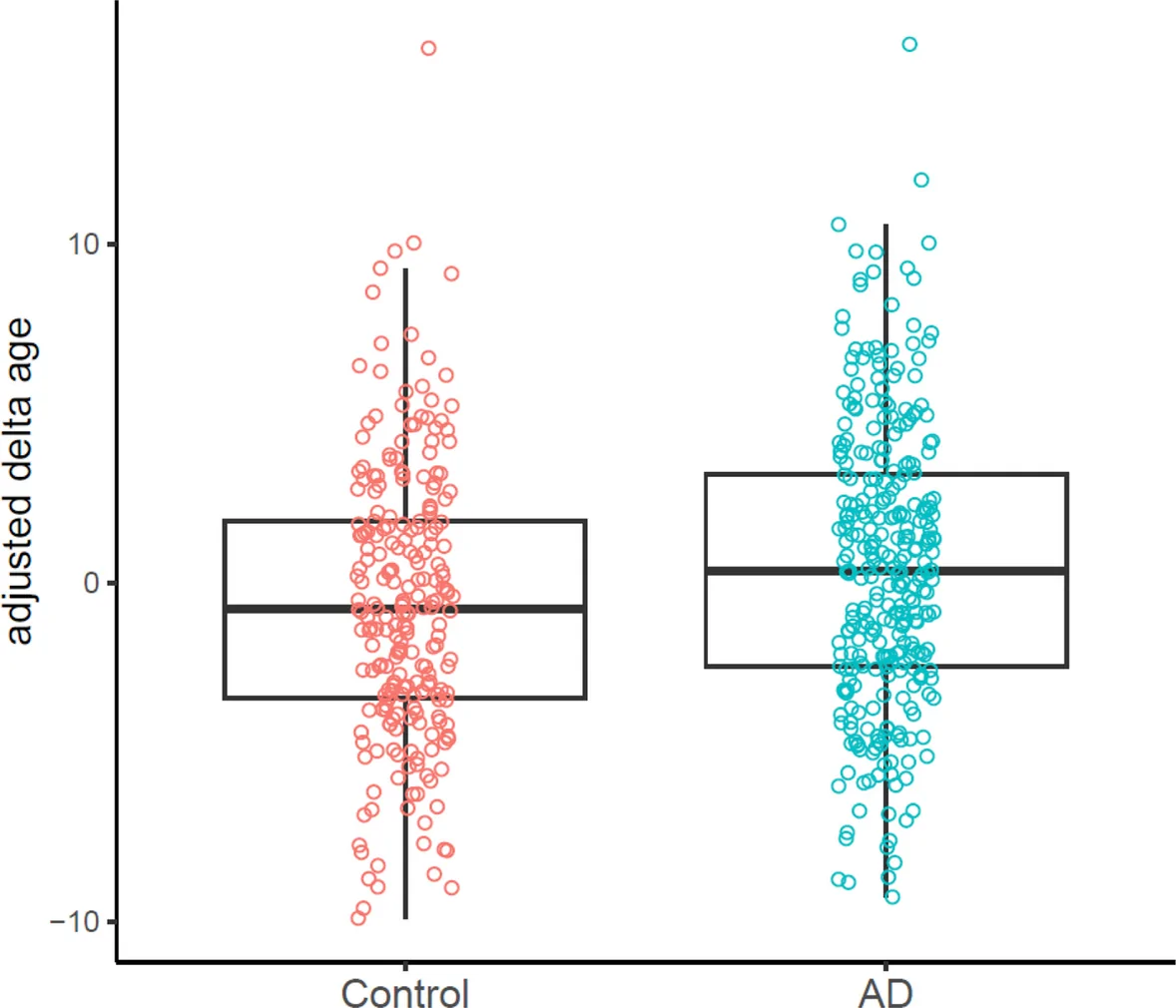
Epigenetic age acceleration by clinical AD diagnosis in ROSMAP. Boxplots show covariate-adjusted delta-age (delta-age = DNAmAge − chronological age) for brain prefrontal cortex samples in ROSMAP. Delta-age was computed using the cortical epigenetic cortical clock, restricted to 100 cortical-clock probes that overlap with the 3264 age-associated CpGs identified in this study. AD status is based on consensus clinical diagnosis at time of death. Points denote individual samples; boxes indicate median and interquartile range; whiskers represent 1.5 × IQR. Higher values correspond to greater epigenetic age acceleration. Adjusted delta-age is the residual delta-age after removing the effects of covariates (age at death, sex, neuronal proportion, batch, and methylation sample plate). In covariate-adjusted linear model, AD cases showed higher adjusted delta-age than controls (*P-*value = 1.40 × 10^–3^)

We next restricted the cortical clock to the subset of 3264 FDR significant aging-associated CpGs identified in the present study, which yielded 100 probes, and recomputed DNAmAge for each subject (Fig. 4). The associations between AD and increased cortical delta-age remained statistically significant (estimate = 1.29, *P-*value = 1.40 × 10^–3^) after adjusting for the same covariates, indicating that the observed acceleration of aging signal is attributable to the age-associated CpGs identified in this prefrontal cortex meta-analysis.

Using the three cerebellum DNAm datasets described in Methods, we applied a cerebellum-specific epigenetic clock [58] to evaluate whether epigenetic age acceleration extends to a region that is relatively spared by AD pathology. For each dataset, we computed cerebellar delta-age and modeled it as a function of AD diagnosis (AD vs. cognitively unimpaired), adjusting for age at death, sex, estimated neuronal proportion, and methylation plate as in the cortical analysis. In all three datasets, the estimated effect of AD on cerebellar delta-age was small and non-significant (estimate = 0.16, 0.31, and 0.85 years of additional DNAm age in AD, with standard error (SE) = 0.38, 0.57, and 1.12; all *P-*values ≥ 0.45; Additional file 2: Fig. S3). An inverse-variance-weighted fixed-effects meta-analysis across the three studies showed no evidence of cerebellar age acceleration in AD (combined estimate = 0.25 years, SE = 0.30, 95% confidence interval (CI) −0.34 to 0.85, *P*-value = 0.40), indicating that, in contrast to cortex, cerebellar epigenetic aging is not significantly accelerated in AD.

### Colocalization analysis nominated genetic variants causal to both ADRD and aging-associated CpG methylation

To further understand the functional roles of the aging-associated CpGs and DMRs in AD, we next performed integrative analyses of aging-associated DNAm with genetic data. First, to identify mQTLs for the significant aging-associated DNAm differences, we searched for mQTLs for these DNAm loci in the brain xQTL database [59]. Among the 3264 aging-associated CpGs (Additional file 1: Table S4) and the 335 CpGs located in significant DMRs (Additional file 1: Table S5), we found that 717 CpGs had 25,661 mQTLs (Additional file 1: Table S16).

As aging and dementia may share common genetic factors, we next evaluated whether these mQTLs overlapped with genetic risk loci implicated in dementia, by comparing them with the genetic variants identified in a recent Alzheimer’s and related dementia (ADRD) meta-analysis [60]. While no mQTLs overlapped with genome-wide significant loci for ADRD, we found 169 SNPs overlapped with genetic variants reaching a suggestive genome-wide significance threshold at *P*-value < 10^–5^ (Additional file 1: Table S17).

Given the observed overlap between the mQTLs and ADRD genetic risk loci, we sought to determine whether the association signals at these loci (variant to CpG methylation levels and variant to ADRD status) were due to a single shared causal variant or distinct causal variants in proximity. To this end, we performed a colocalization analysis using the method described by Giambartolomei et al. [62]. The results strongly suggested [89] (i.e. PP3 + PP4 > 0.90, PP4 > 0.8 and PP4/PP3 > 5) that 4 genomic regions included a single causal variant common to both phenotypes (i.e., ADRD status and CpG methylation levels). The CpGs associated with these causal variants are located in the *CHRNE*, *IDUA, KCNN4*, and *MLNR* genes (Additional file 1: Table S18).

### Brain-to-blood DNAm correlation analysis identified aging-associated brain DNAm with concordant cross-tissue changes

To identify aging-associated DNA methylation (DNAm) with the potential to serve as biomarkers, we next evaluated brain-to-blood DNA methylation correlations of the aging-associated DNAm. Among the 3264 significant individual CpGs associated with aging and 335 CpGs located in aging DMRs, DNAm at 152 CpGs showed significant brain-to-blood correlations (*FDR* < 0.05) (Additional file 1: Table S19). Among the 152 CpGs with significant brain-to-blood correlations, all but one showed a significant positive association, corroborating previous analyses [32] that observed a significant negative correlation between the brain and blood is relatively rare. The positive correlations ranged from 0.335 to 0.706. Notably, 22 (14.5%) of the 152 CpGs with significant brain-to-blood correlations are located on the *HOX* gene cluster (*HOXA2, HOXA4, HOXA5*) on chromosome 7. These regions with consistent cross-tissue epigenetic modifications may indicate important shared regulatory roles across tissues during aging.

### Validation of concordant brain DNAm differences in aging and AD neuropathology (Braak stage) using independent datasets

As mentioned above, we identified 249 CpGs hypermethylated in both aging and AD neuropathology (Braak stage), and 85 CpGs hypomethylated in both conditions (Fig. 3). To further validate these 334 (249 + 85) concordant brain DNAm differences, we examined their presence in previous AD studies using the MIAMI-AD database (https://miami-ad.org/) [63]. In this comparison, we made sure all referenced datasets were independent of ROSMAP and BDR. We found that among the 334 CpGs, 325 (97.3%) had nominal significant *P-*values in earlier studies, with consistent directions of change (Additional file 1: Table S20). Similarly, of the 15 DMRs with concordant aging and AD changes in Fig. 3, all included one or more CpGs that reached nominal significance in previous analyses (Additional file 1: Table S21). Notably, many of these prior studies analyzed DNAm data from various brain regions (e.g., temporal cortex) or even whole blood, consistent with the observation that the majority of age-associated DNAm differences are not cell-type specific [85, 90]. For example, PRC2-marked sites, where many of the aging-associated CpGs we identified are located, have been shown to undergo hypermethylation with aging, independent of tissue or cell type [91–94].

### Concordant brain DNAm is associated with AD progression in out-of-sample validation

We next evaluated the feasibility of using concordant DNAm changes associated with both aging and AD neuropathology as potential biomarkers of clinical AD progression. First, we overlapped the 374 CpGs showing concordant aging- and AD Braak stage-associated DNAm changes (343 individual CpGs and 40 CpGs within concordant DMRs) with the 152 CpGs showing significant brain-to-blood correlations, which yielded 33 candidate CpGs. We then performed out-of-sample validation in ADNI, as described in Methods. For each subject, we analyzed the earliest available (baseline) DNAm sample measured in whole blood. These subjects were followed an average of 5.39 ± 2.94 years, with follow-up durations ranging from 0.44 to 11.71 years. By their last visit, 64 (30.0%) cognitively unimpaired subjects had progressed to MCI or AD, and 131 (40.3%) MCI subjects had progressed to AD.

For each of the 33 candidate CpGs that showed concordant DNAm changes with aging and AD neuropathology in cortex as well as significant brain-blood DNAm correlations, we used Cox regression models to test whether baseline DNAm beta values were associated with disease progression (CU to MCI/AD or MCI to AD). The models were adjusted for age, sex, *APOE* ε4 allele status, years of education, and baseline MMSE score. Multiple comparison correction was performed across these 33 candidate CpGs. At 5% FDR, we identified one CpG, cg10752406 located in the *AZU1* gene promoter, that was significantly associated with progression to the next disease stage (*P-*value = 0.0012, FDR = 0.0397) (Table 3). The covariate-adjusted survival curves derived from the multivariable Cox model showed that subjects in the highest and lowest tertiles of methylation for cg10752406 had significantly different survival probabilities over time (Additional file 2: Fig. S4). Although survival probability decreases in both groups over time, subjects with lower methylation consistently show a lower survival probability, indicating higher risk of AD progression. By design, cg10752406 also showed a significant brain-blood DNA methylation correlation, and was significantly associated with both aging and increased Braak stage (Additional file 1: Table S23).

**Table 3:**
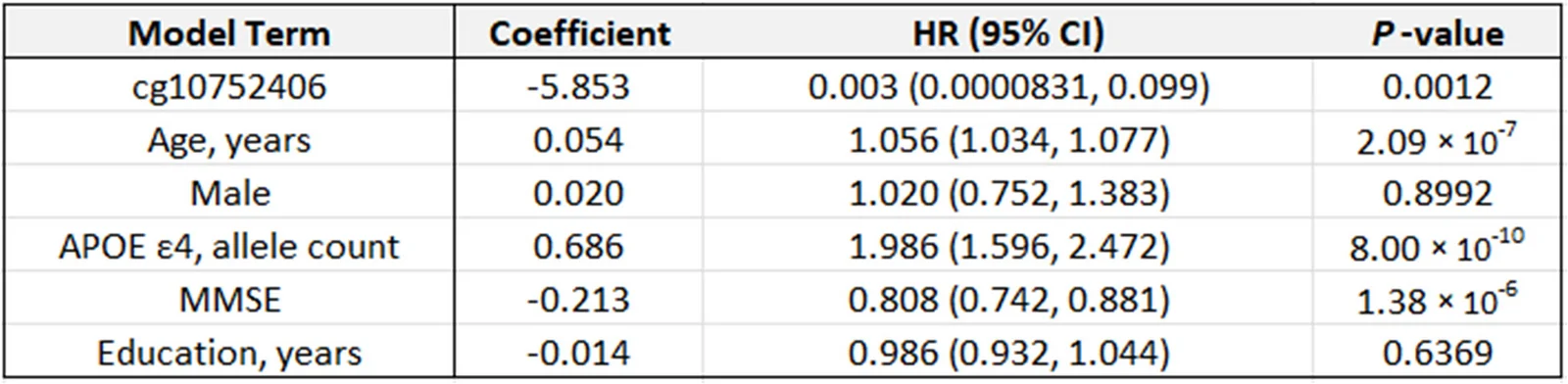
Results from Cox regression model evaluating the association between DNA methylation at cg10752406 and disease progression (CU to MCI or AD, MCI to AD) in 538 ADNI subjects, adjusted for age, sex, *APOE ε4*, baseline MMSE, and education. Significant association was observed for cg10752406 (estimate = −5.853, *P*-value = 0.0012, FDR = 0.0397), indicating lower methylation at this CpG is associated with increased risk for AD progression

*Abbreviations*: *CU* Cognitively unimpaired, *MCI* Mild cognitive impairment, *AD* Alzheimer’s disease, *HR* Hazard ratio, *CI* Confidence interval, *MMSE* Mini-Mental State Examination

## Discussion

We performed a comprehensive meta-analysis of two large cohorts of prefrontal cortex brain samples to prioritize consistent DNA methylation differences involved in aging. Consistent with previous brain sample studies [65, 66], we found the majority of aging-associated CpGs were hypermethylated with increased age. Enrichment analysis showed that these hypermethylated aging-associated CpGs were significantly enriched in promoter regions and CpG islands, but under-represented in the open sea, shore, and gene body. In contrast, hypomethylated CpGs were enriched in open sea, shelf, and gene body regions, but depleted in CpG islands and promoter regions. Furthermore, comparison with chromatin state annotations generated by the Roadmap Epigenomics project [54, 79] showed that hypermethylated aging-associated CpGs were enriched in genomic regions with potential regulatory activity but was maintained in poised states, such as bivalent enhancers and repressed polycomb regions. On the other hand, hypomethylated aging-associated CpGs were more likely to be found in distal regulatory regions, such as enhancers. Consistent with these findings, our integrative analysis with ENCODE data revealed hypermethylated CpGs, but not hypomethylated CpGs, were also enriched in binding sites of several transcription factors and chromatin proteins, including EZH2 and SUZ12, which are subunits of the PRC2 protein complex, as well as the insulator protein CTCF.

To evaluate whether the aging-associated DNAm patterns we identified in late-life brain tissue are unique to the brain or reflect more systemic aging processes, we compared our results to those from a recent large-scale blood EWAS of aging by Seale et al. (2024), which meta-analyzed 56 datasets comprising over 32,000 human blood methylomes [95]. In blood, approximately two-thirds of age-associated CpGs are hypomethylated with age [95], while in this brain cortex analysis, we found the majority of age-associated CpGs (~ 85%) are hypermethylated. Despite this difference, both tissues showed strong enrichment of age-associated hypermethylation at poised regulatory regions, such as bivalent domains and Polycomb-repressed chromatin.

Bivalent domains, marked by both activating (H3K4me3) and repressive (H3K27me3) histone modifications, keep developmental genes in a transcriptionally poised state [96, 97]. Age-related hypermethylation in these regions may convert reversible repression into a more stably repressed state, reducing plasticity and the capacity to maintain or restore cell identity [95]. Because bivalent/PRC2-marked elements occur across many somatic cell types and show similar age-enrichment patterns across tissues [98], hypermethylation here may represent a shared epigenetic hallmark of aging. Consistent with this, our prior AD neuropathology meta-analysis found that hypermethylated CpGs associated with increasing Braak stage were enriched in bivalent and Polycomb chromatin states [19]. Together with the enrichment we observe for brain aging and that reported in blood [95], this points to bivalent and Polycomb domains as recurrent targets of age- and AD-related epigenetic remodeling [99].

The predominance of PRC2 (EZH2/SUZ12) enrichments in non-central nervous system (CNS) tracks is consistent with well-described age-related hypermethylation at Polycomb targets that are broadly shared across tissues [91]. The presence of CTCF enrichment in CNS-relevant contexts (astrocytes, human brain microvascular endothelial cells (HBMEC), neural lines) and EZH2 enrichment in NH-A (normal human astrocytes) supports relevance to glial/endothelial regulation in brain. However, LOLA relies on public ChIP-seq compendia that are skewed toward peripheral or immortalized cell types (e.g., ENCODE, Roadmap); thus, these overlaps should be interpreted as regulatory context signals rather than strict tissue specificity. Future work leveraging brain region- and cell-type-specific epigenomes will provide additional insights into the tissue-specificity of the observed enrichments.

Consistent with a previous study [65], we found nearly all DNAm differences significantly associated with both age and AD Braak stage in prefrontal cortex have directionally concordant effect sizes. We further evaluated epigenetic age acceleration in the cortex and found that delta-age (DNAmAge − chronological age) was significantly elevated in ROSMAP subjects with clinically diagnosed AD compared to cognitively unimpaired subjects, after adjusting for age at death, sex, neuronal proportion, batch, and methylation plate. These results extend prior reports associating epigenetic age acceleration with AD neuropathology in the cortex by demonstrating that the association persists in clinical AD cases, and when the cortical clock is restricted to the aging-associated CpGs identified in this study, indicating that acceleration signal in AD is attributed to age-associated CpGs in the cortex.

In contrast to cortical regions, we did not observe robust epigenetic age acceleration in the cerebellum when comparing AD cases with controls. This result is consistent with prior work showing that epigenetic clocks generally do not show cerebellar age acceleration in the presence of AD pathology [100, 101]. Accumulating evidence indicates epigenetic clocks capture distinct aging trajectories across brain regions and cell types, rather than a uniform global aging signal [101–103]. In particular, cell-type–specific DNAm clocks indicate clear biological age acceleration in AD brains, with the strongest effects seen in glial clocks in the temporal lobe and weaker but statistically significant acceleration in neuronal clocks [102]. Similarly, single-nucleus transcriptomic aging clocks demonstrate significant age acceleration in glial cell types, and trends toward accelerated aging in neuronal populations in AD brains [103]. These findings indicate that AD is associated with intrinsic aging of specific cell types in cortical circuits, rather than a uniform acceleration across all brain regions. In this context, the absence of strong cerebellar age acceleration in our data does not argue against accelerated brain aging in AD; instead, it supports a regional and cell-type-selective model in which aging-related epigenetic programs are most altered in cortical glia and neurons.

Although we did not analyze epigenetic age acceleration in blood in the present study, prior work with blood-based clocks provides important context for interpreting our findings. Blood DNAm age acceleration has shown mixed associations with cognitive decline and dementia: some studies report that higher acceleration for second- or third-generation blood clocks (e.g., GrimAge, PhenoAge, DunedinPACE) is associated with poorer cognition, incident dementia, or faster disease progression [104–106], while other cohorts observe little or no association [107, 108]. A recent systematic review likewise concluded that evidence for blood DNAm age acceleration as a biomarker of dementia and related outcomes is modest and inconsistent [109]. Overall, these findings suggest that blood-based age acceleration is at best a modest and context-dependent marker of dementia risk, supporting the view that cortical clocks may capture aspects of aging that are not fully reflected in peripheral blood.

Beyond the epigenetic clocks, we found that many of the aging-associated DNAm differences in prefrontal cortex identified in this study overlap with CpGs associated with AD neuropathology (Braak stage) in our prior meta-analysis and have also been previously implicated in AD. Among the 334 CpGs significantly associated with both aging and AD Braak stage, 97.3% had previously been linked to AD or AD neuropathology with consistent directional changes in prior studies. Importantly, these findings also pointed to new therapeutic targets. For example, we observed extensive hypermethylation at genes belonging to the HOX family. Previously, aberrant methylation on the *HOXA* gene cluster on chromosome 7 was shown to be associated with AD neuropathology in multiple AD EWAS datasets [22, 110, 111]. DNAm at the *HOX* genes has also been observed to interact with the protein complex PRC2, which regulates neuronal lineage specification and maintains neuronal functions. Importantly, PRC2 silences genes involved in neurodegeneration, and its deficiency leads to the de-repression of developmental regulators such as the HOX gene clusters, resulting in progressive and fatal neurodegeneration in mice [99]. Encouragingly, recent studies have demonstrated that lifestyle factors may partially reverse age-related epigenetic changes. Increased physical activity, for example, has been observed to be associated with hypomethylation at *HOXA3* and *HOXB1* genes in human muscles [72].

Moreover, our out-of-sample validation using the ADNI dataset focused on a set of 33 candidate CpGs that showed concordant brain DNAm differences in aging and AD neuropathology (Braak stage) in prefrontal cortex, as well as significant brain-blood methylation correlations. Within this candidate set, one CpG, cg10752406 located in the promoter of *AZU1*, was significantly associated with AD progression, even after controlling for age, sex, *APOE* ε4, years of education, and baseline MMSE score. The *AZU1* gene, also called *CAP37,* encodes a neutrophil granule protein involved in inflammation and host defense [112]. In AD, the mRNA expression levels of *CAP37* is elevated, possibly due to increased amyloid beta and proinflammatory cytokines that activate microglial cells [113, 114]. This candidate-based finding suggests that cg10752406 merits further study in larger cohorts to evaluate its potential contribution as a peripheral biomarker for AD.

Our findings complement and extend those of Pellegrini et al. [65], who conducted a meta-analysis of DNAm across multiple brain regions, examining associations with age, sex, and clinical AD diagnosis. While both Pellegrini et al. (2021) and our studies observed concordant age- and AD-related methylation changes, our primary AD-related results are based on neuropathologic Braak stage rather than clinical diagnosis, which more closely reflects the underlying pathology. There are several additional important distinctions. First, our analysis focused specifically on late-life aging (> 65 years) in the prefrontal cortex using samples with minimal AD neuropathology (Braak stage 0-II), to isolate aging effects from early disease-related changes. In contrast, Pellegrini et al. (2021) analyzed a broader age range and defined AD and control groups based on clinical diagnosis, which may be less precise in distinguishing true AD pathology. Second, the frontal cortex meta-analysis in Pellegrini et al. (2021) included fewer samples, with 187 AD and 112 control samples in total, which identified only 14 AD-associated CpGs. Our study compared aging-associated results with a large-scale AD neuropathology meta-analysis of over 1000 prefrontal cortex samples classified by neuropathological Braak staging [19], which identified 2767 AD Braak-associated CpGs, providing a much richer set of signals for overlap and functional analyses. Finally, our work incorporated several additional components that were not present in Pellegrini et al. (2021), including chromatin-state analyses, integration with brain mQTL and ADRD GWAS data, evaluation of brain-to-blood DNAm correlations to nominate peripheral biomarker candidates, and longitudinal validation in the ADNI cohort showing that one concordant CpG (*AZU1*, cg10752406) predicts AD progression.

The considerable overlap we observed between age‑associated CpGs and those altered in relation to AD neuropathology (Braak stage) should not be taken to mean that these epigenetic changes are deleterious. Because bulk postmortem tissue from AD brains is enriched for cells that have survived neurodegeneration, the DNA profiled in the AD neuropathology study to identify Braak stage-associated DNAm is likely drawn from relatively resilient neurons and glia. Moreover, these sites did not overlap with AD susceptibility loci identified by recent GWAS, arguing against their primary role in disease initiation. Instead, the shared methylation landscape may reflect adaptive or stress‑response programs that help certain brain cells withstand the challenges of aging and pathology. Future studies using single cell methylome profiling and longitudinal designs are needed to identify the specific cell types involved in these methylation changes and to determine whether they contribute to cognitive decline or instead reflect resilience.

This study has several limitations. First, we analyzed DNA methylation measured in bulk prefrontal cortex brain samples. To reduce confounding effects due to different cell types, we included estimated cell-type proportions as covariate variables in all our analyses. Although our results and previous studies have demonstrated that the majority of aging-associated DNAm is relatively robust across various tissues, future studies utilizing single-cell technology would provide more refined insight into the specific cell types affected by the aging-associated DNAm differences discovered in this study. Second, the Illumina arrays used by the studies analyzed in this meta-analysis cannot distinguish 5-methylcytosine (5mC) from 5-hydroxymethylcytosine (5hmC). Meta-analysis strategies similar to the one employed here could be applied to large datasets that discriminate between 5mC and further oxidized DNA modifications. Third, we focused our analyses on DNAm measured in the prefrontal cortex, a region significantly involved in aging and AD, partly due to the availability of large, high-quality DNAm datasets. It has been observed that there is a substantial correlation between DNAm in different brain regions [31]. Moreover, previous studies have found the largest number of age-associated and AD neuropathology-associated brain DNAm changes in the frontal cortex compared to other brain regions (e.g., temporal cortex, entorhinal cortex, cerebellum) [31, 45, 65]. Other brain regions, such as temporal cortex and entorhinal cortex, can be studied similarly as more data become available. Fourth, while Berger and colleagues identified aging-dysregulated histone modifications in AD [115], we did not observe similar DNA methylation patterns in our study. This discrepancy could be due to the limited coverage of the methylation arrays, which mainly focus on gene promoters and CpG islands. A previous study using next-generation sequencing technology found a number of hypomethylated DMRs associated with aging, with the majority lying outside of CpG islands and regions targeted by arrays [116]. Future studies with whole-genome bisulfite sequencing (WGBS) are needed to investigate the global landscape of aging DNAm in AD more comprehensively. Finally, we analyzed only DNAm samples from non-Hispanic white subjects. Future studies that investigate DNA methylation in large, multi-ethnic cohorts are needed.

## Conclusions

In summary, we conducted a comprehensive meta-analysis to identify DNAm differences in the prefrontal cortex consistently associated with aging across two large brain sample cohorts. Our analyses revealed numerous robust DNAm differences associated with aging, highlighting key genes such as *ELOVL2*, *ISM1*, and *KLF14*, all of which have been implicated in various aging processes. We also observed significant overlaps between aging-associated DNAm changes and those involved in AD, as well as accelerated aging in AD cases compared to cognitively unimpaired controls, supporting the hypothesis that aging and AD share common molecular underpinnings. Furthermore, our analyses using independent datasets confirmed both the validity of the concordant brain DNAm differences in aging and AD neuropathology and their potential to predict AD progression. Taken together, these findings underscore the importance of aging as a critical factor in the pathogenesis of AD and suggest that age-related epigenetic modifications may offer a promising avenue for AD biomarkers.

## Supplementary Information

Additional file 1: Tables S1-S23. Supplementary tables containing cohort and dataset summaries, quality-control information, significant CpGs and DMRs, enrichment results, comparisons with AD neuropathology, mQTL and ADRD GWAS colocalization results, brain-blood DNAm correlations, MIAMI-AD validation, and ADNI progression analyses.

Additional file 2: Fig. S1-S4. Supplementary figures showing BDR low-pathology classification, matched-sample comparisons, cerebellar epigenetic age acceleration analyses, and ADNI covariate-adjusted survival curves.

## Data Availability

All datasets analyzed in this study are publicly available or available through established data-access procedures. ROSMAP data can be accessed from the AD Knowledge Portal [26] (accession: syn3157275) and are described in prior publications [24]. BDR DNAm data can be accessed from the Gene Expression Omnibus database (GEO; accession: GSE197305) and are described in Smith et al. [45]. The London matched brain-blood dataset can be accessed from GEO (accession: GSE59685) and is described in prior publications [31, 32]. The ADNI dataset can be accessed from http://adni.loni.usc.edu, and the ADNI DNAm data are described in Vasanthakumar et al. [33]. The three cerebellum DNAm datasets can be accessed from GEO (accessions: GSE134379, GSE105109, and GSE59685) and are described in prior publications [29–31]. The scripts for the analysis performed in this study can be accessed through the project GitHub repository [117].
