# Supplementary material for: From aging to Alzheimer’s disease: concordant brain DNA methylation changes in late life
Source: Genome Med. 2026 Jun 16;18:126. doi: 10.1186/s13073-026-01698-8 (PMC13505181; doi:10.1186/s13073-026-01698-8)
Supplement: Supplementary file 1 [file 13073_2026_1698_MOESM2_ESM.pdf]

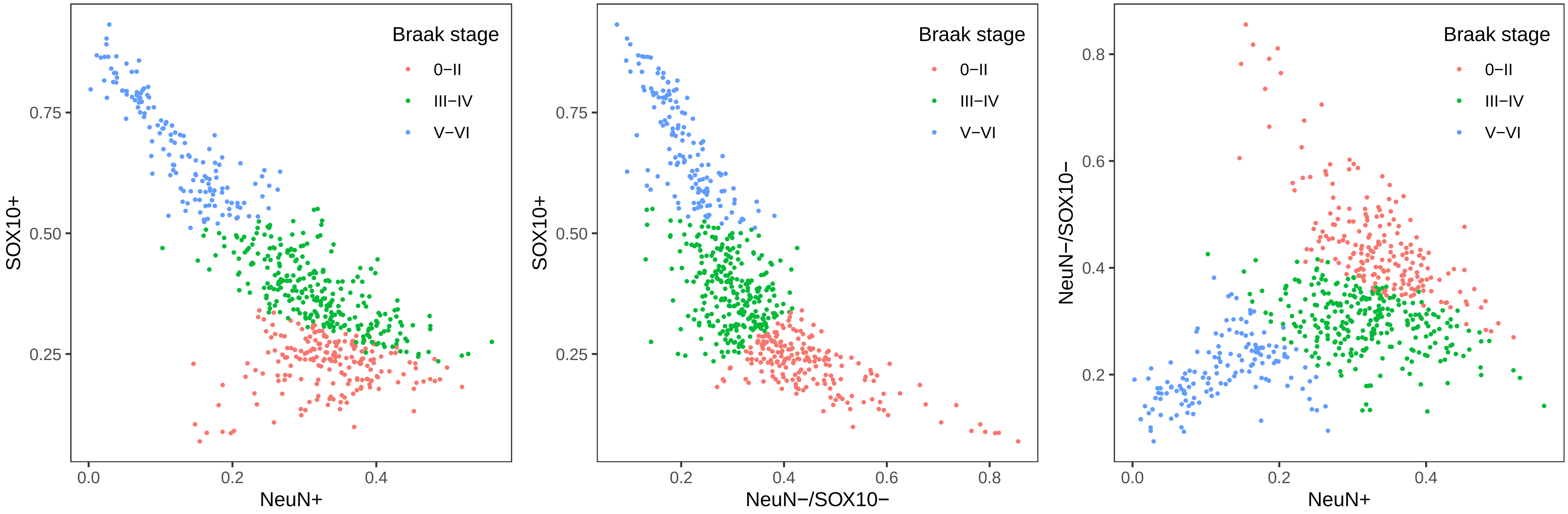

Figure S1 K-means (k = 3) clustering of bulk cortex brain samples in the BDR cohort using estimated relative cell type proportions of NeuN+, SOX10+, and NeuN-/SOX10-. These results are consistent with those in Shireby et al. (2022) (PMID: 36153390), showing that an increase in tau pathology corresponds to a decrease in the proportion of NeuN+ (neuronal) cells, a reduction in NeuN-/SOX10- (microglial/astrocytic) proportions, and an increase in SOX10+ (oligodendrocytic) proportions in the brain cortex. The current study only used samples in the Braak stage 0-II group.

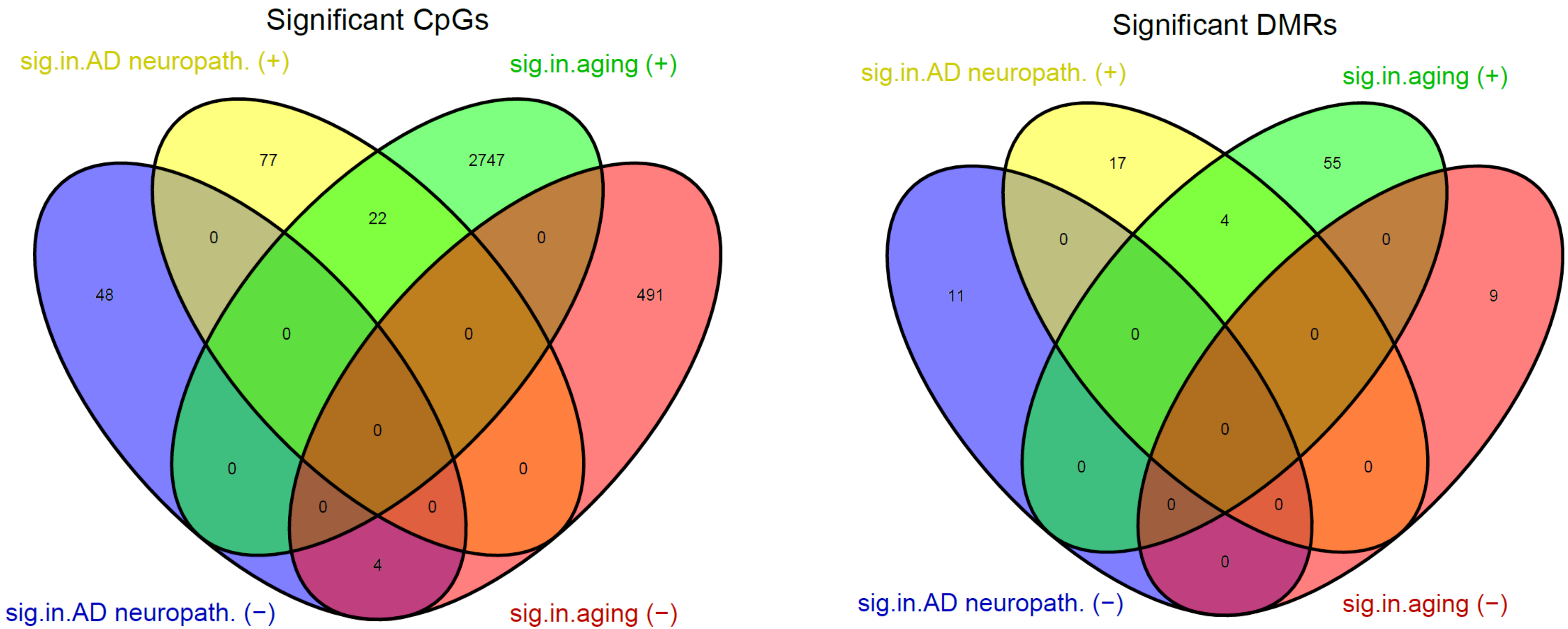

Figure S2 Comparison of aging-associated DNA methylation (DNAm) and AD Braak stage-associated DNAm (from matched samples analysis) shows that all of the CpGs and DMRs significantly associated with both age and AD Braak stage showed concordant direction of change. In (a), among the overlapping CpGs, 22 out of 22 CpGs that showed hypermethylation with increasing age also showed hypermethylation with advancing Braak stage, and 4 out of 4 CpGs that showed hypomethylation with increasing age also showed hypomethylation with advancing Braak stage. Similarly, in (b), all 4 overlapping DMRs have concordant direction of change in aging and AD Braak stage. The significant AD Braak-associated DNAm were obtained from Supplementary Data 6 and 7 in Zhang et al. (2020) Nat. Comm. (PMID: 33257653). **Abbreviations** sig.in.aging (+): significantly hypermethylated in aging; sig.in.aging (-): significantly hypomethylated in aging; sig.in.AD neuropath. (+): significantly hypermethylated with increased AD Braak stage; sig.in.AD neuropath. (-): significantly hypomethylated with increased AD Braak stage.

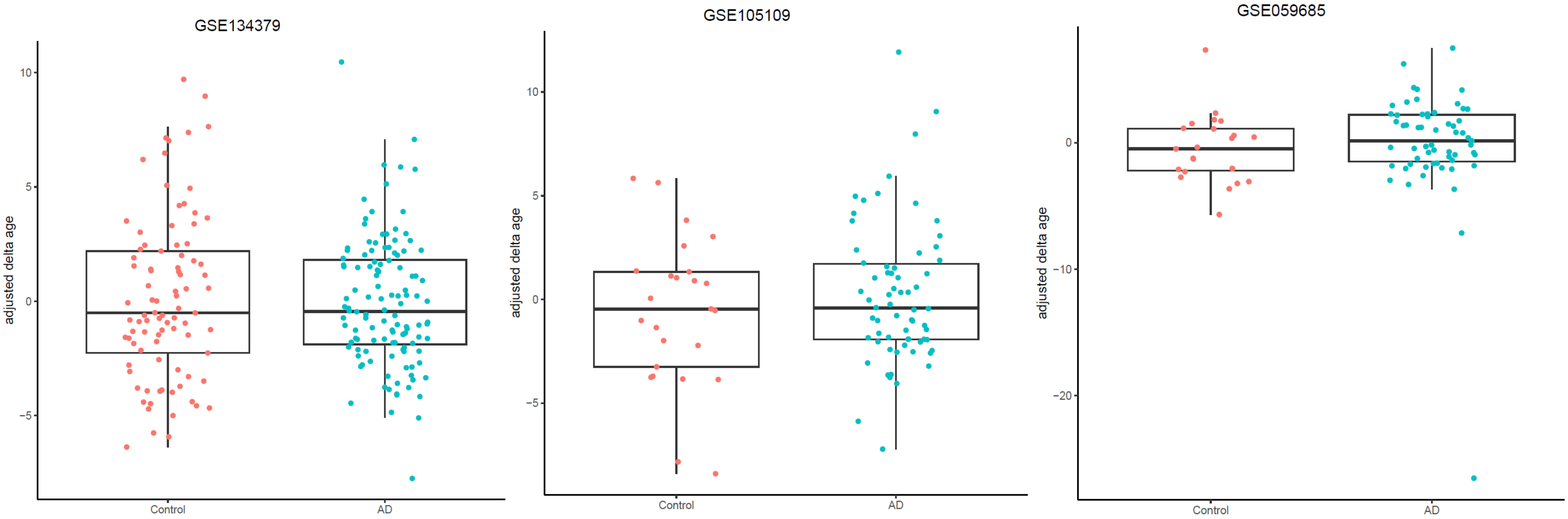

Figure S3 Epigenetic age acceleration in brain cerebellum by clinical AD diagnosis. Boxplots show covariate-adjusted delta age (delta age = DNAmAge − chronological age) for brain cerebellum samples in three DNA methylation datasets from GEO database. Delta age was computed using the cerebellum-specific epigenetic clock (PMID: 37597113). Points denote individual samples; boxes indicate median and interquartile range; whiskers represent 1.5×interquartile range. Higher values correspond to greater epigenetic age acceleration. Adjusted delta age is the residual delta age after removing the effects of covariates (age at death, sex, neuronal proportion and methylation plate). In all three datasets, cerebellar delta-age was not significantly different between AD and control samples.

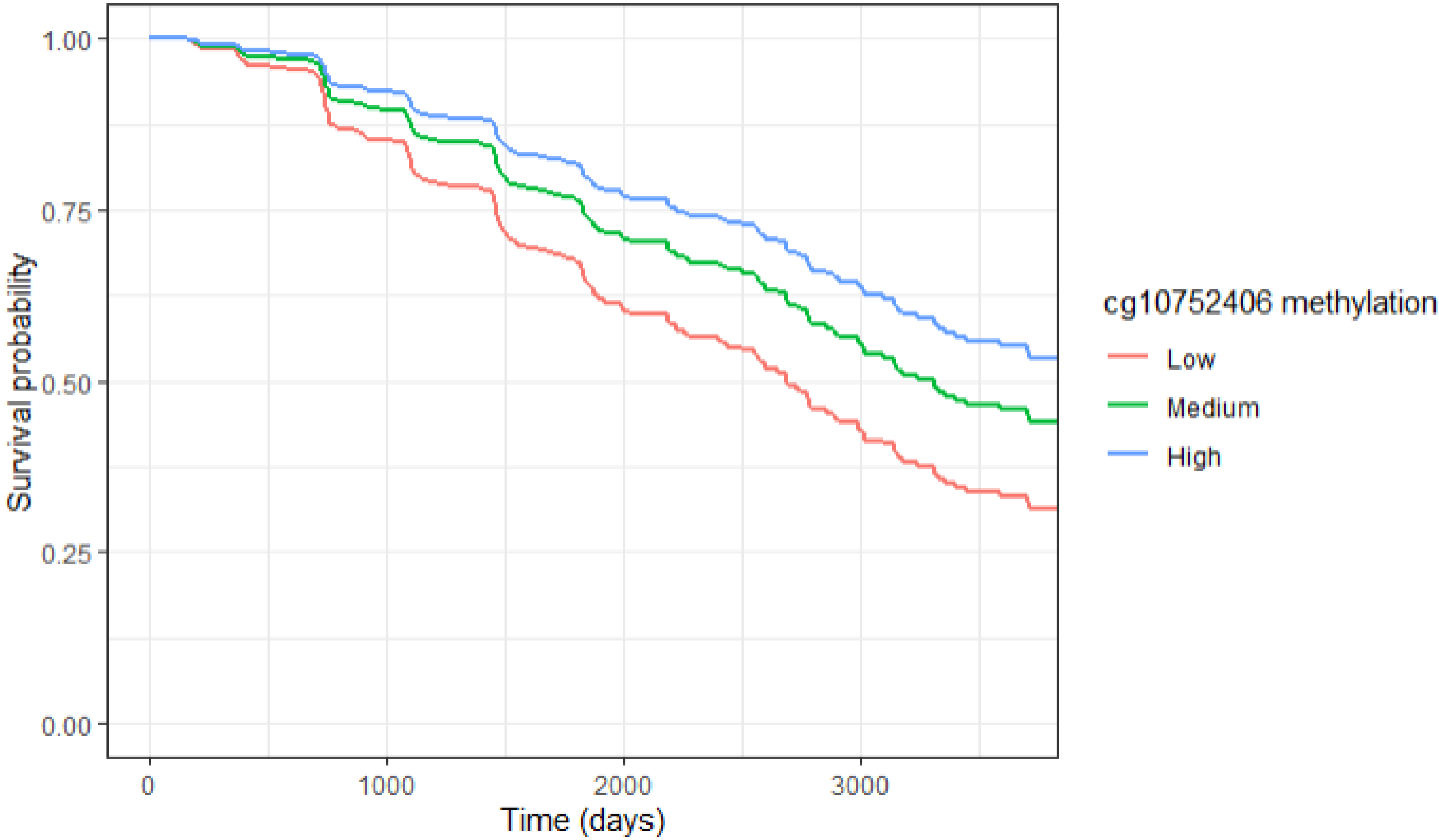

Figure S4 Covariate-adjusted survival curves for AD progression by cg10752406 methylation tertiles in ADNI. Model-based survival curves were estimated from a multivariable Cox proportional hazards model of time to progression to a more severe diagnostic stage (cognitively normal or MCI to AD), with baseline whole-blood DNAm at cg10752406 entered as tertiles (Low, Medium, High) and adjusting for age at visit, sex, APOE ε4 carrier status, years of education, and baseline MMSE score. Curves were obtained using ggadjustedcurves function with covariate values averaged over the study sample, so that differences in survival probabilities reflect the association of cg10752406 methylation with progression risk after covariate adjustment.
